# Molecular Detection of *Aspergillus*: Application of a Real-Time PCR Multiplex Assay in Tissue Samples

**DOI:** 10.3390/jof6010011

**Published:** 2020-01-10

**Authors:** Raquel Sabino, Helena Simões, Cristina Veríssimo

**Affiliations:** Reference Unit for Parasitic and Fungal Infections, Department of Infectious Diseases, National Institute of Health Dr. Ricardo Jorge, Av. Padre Cruz, 1649-016 Lisbon, Portugal; helena.simoes@insa.min-saude.pt (H.S.); cristina.verissimo@insa.min-saude.pt (C.V.)

**Keywords:** *Aspergillus*, invasive fungal infections, real-time PCR, molecular diagnosis, azole-resistance

## Abstract

Diagnosis of invasive fungal infections is complex, and the lack of standardization of molecular methods is still a challenge. Several methods are available for the diagnosis of invasive aspergillosis, but their effectiveness will depend on the studied population, the patients’ comorbidities, and the use of mold active prophylaxis, among others. The ability to determine the identity of the infecting *Aspergillus* species, and to detect mutations conferring specific resistance patterns directly from DNA extracted from the biological product, is an advantage of nucleic acid testing compared with antigen-based assays. In this study, we present laboratory cases where the diagnosis of aspergillosis was performed using a real-time multiplex PCR for the detection of *Aspergillus* DNA in tissue samples, showing its usefulness as one more tool in the diagnosis of aspergillosis in tissue samples. *Aspergillus* real-time multiplex PCR was also used to detect azole-resistance in some cases. In the majority of the PCR positive cases, cultures remained negative after 60 days. The PCR assay directed to *Aspergillus* gave positive signals for *Aspergillus fumigatus* sensu stricto. Results were confirmed by panfungal PCR, followed by sequencing, revealing 100% homology with *Aspergillus fumigatus* sensu stricto. Mutations conferring azole resistance were not detected.

## 1. Introduction

Invasive fungal infections have significantly increased due to advances in medical care in the at-risk immunocompromised population [1]. Changes in the spectrum of the fungal infections associated with new risk factors and the emergence of resistant fungi highlight the need for continuous updates on knowledge of the epidemiology of fungal infections [2]. 

*Aspergillus* is the filamentous fungi more frequently associated with invasive fungal infection. According to the LIFE organization, more than 30 million patients are at risk for invasive aspergillosis and about 300,000 patients will develop the disease annually [3]. 

Patients at higher risk of acquiring invasive aspergillosis (IA) often suffer from severe granulocytopenia (i.e., leukemia and bone marrow or solid organ transplanted patients). Also, long-term corticosteroid use, diabetes, major burns, and recent major surgeries are considered as predisposing risk factors. Early diagnosis of invasive aspergillosis (IA) is a challenge and should be based on the integration of clinical, radiological, and microbiological data.

Invasive aspergillosis is difficult, and treatment (often too late) is only partially effective. *Aspergillus* spp. causes severe infections with a mortality rate that may reach 50% if treated and more than 99% if not [3]. Diagnosis of proven IA relies on positive cultures obtained from sterile sites [4] or on histopathological observation of fungal elements in the tissue which requires semi-invasive or invasive procedures such as bronchoalveolar lavage (BAL) or computed-tomography (CT)-guided needle biopsy [5]. Fungal elements (hyphae) display the proof of an infection if present in primarily sterile specimens, independent of culture results. Microscopy should be performed, preferably using optical brighteners and histopathology using Gomori’s methenamine silver stain or periodic acid-Schiff [6].

There are, however, disadvantages associated with these methods: cultures may remain negative after weeks of incubation, not allowing species identification of the etiological agent nor the determination of the antifungal susceptibility profile. On the other hand, histopathological procedures require a high level of experience of the laboratory technician and do not allow the identification of a species by the fungal structures observed in the tissue sections.

Further concerns regarding aspergillosis have been rising recently: azole resistance has been increasing in prevalence in human clinical isolates of *A. fumigatus*. In some instances, acquired resistance may be driven by antifungal selection in patients receiving long-term prophylaxis/therapy. Nevertheless, many azole-resistant strains originated in the environment due to selection by azole fungicides used in agriculture. It has been shown that azole-resistant *A. fumigatus* isolates that harbor specific *CYP51A*-mediated resistance mechanism may show resistance to one azole or all classes of azoles, depending on the mutation [7].

*CYP51A* target enzyme alterations are the most commonly encountered resistance mechanisms in *A. fumigatus* isolates. The target enzyme changes include nonsynonymous single-nucleotide mutations, inducing amino acid substitutions at hotspots, such as G54, M220, P216, G138, and G448, or nonsynonymous resistance mutations in combination with tandem repeat (TR) in the gene promoter region, such as TR34/L98H and TR46/Y121F/T289A [8,9]. Of particular concern are the two resistant *A. fumigatus CYP51A* genotypes: TR34/L98H and TR46/Y121F/T289A, which have been associated with environmental triazole fungicide use rather than previous patient exposure to antifungals. Importantly, these genotypes confer resistance to itraconazole or voriconazole, or both [10]. Genetically, the high resistance is based on a tandem repeat in the promoter region in combination with one or more specific point mutations in the coding region of the *CYP51A* gene. A point mutation or tandem repeat alone yields low levels of resistance; only a combination of both results in the fully resistant phenotype [11]. 

An early diagnosis and prompt initiation of appropriate antifungal therapy are imperative and essential for a favorable clinical outcome [12]. Thus, the reduction of mortality among patients with IA must be accompanied by research that allows earlier diagnosis by traditional and non-cultural-based molecular tests as well as the development of new strategies for the detection of antifungal resistance. 

Serum and BAL galactomannan assays are recommended as markers for the diagnosis of invasive aspergillosis; PCR should be considered in conjunction with other diagnostic tests. Antifungal treatment decreases GM sensitivity. Pathogen identification to species complex level is strongly recommended for all clinically relevant *Aspergillus* isolates [6].

Antigen-based assays in BAL are strongly recommended for the diagnosis of pulmonary IA in all patient groups. When other biological products are used (blood, cerebrospinal fluid, lung biopsies), the recommendation for galactomannan detection in the diagnosis of IA or its prospective screening is variable (from A to D class recommendation), depending on the patients’ comorbidities and the use (or not) of mold active prophylaxis.

The ESCMID-ECMM-ERS guideline strongly supports (class A recommendation) the use of PCR and antigen-based assays (galactomannan) in BAL to the diagnosis of IA in haematopoieic stem cell transplanted patients [13]. A constituent of the cell wall of many species and genera of fungi, (1-3)-b-D-glucan (BDG), is released into body fluids in association with fungal infection. A limited role is given for the exclusive testing of the BDG in diagnosing IA (BII); however, the combination with GM or PCR improves specific detection [14]. Despite being a very promising tool, the sole usage of PCR on blood or serum in this same group of patients and the ones with hematological malignancies has a B recommendation. On the other hand, molecular diagnosis using a broad-range PCR on biopsies with visible hyphae to detect and specify a fungus is a strongly supported recommendation (Class A) since several studies demonstrated high sensitivity (>90%) and high specificity (99%) whereas an *Aspergillus* PCR is, until now, moderately supported [13]. *Aspergillus* PCR has been applied mostly to blood and BAL fluid. For both sample types, a combination with other biomarkers increases the likelihood of IA [13].

A variety of research-use-only and/or CE-marked commercial kits for the direct detection of *Aspergillus* nucleic acid have been described. The performance of these assays varies on the basis of specimen type and patient population and the manner in which infection status was determined in the study [15]. 

The ability to determine the identity of the infecting species (and consequently possible antifungal patterns), and to detect mutations conferring specific resistance patterns, especially directly from DNA extracted from the biological product (without the need of a positive culture), is the differential advantage of nucleic acid testing compared with antigen-based assays.

Even though *Aspergillus fumigatus* sensu stricto is the most common species that causes IA, in recent years, there has been an increase in the number of species in the different sections, which makes the diagnosis of this invasive fungal disease a great challenge [16]. Therefore, PCR based detection and identification of *Aspergillus* in tissue samples, together with the detection of possible mutations that confer azole resistance to azoles, enable the mycology laboratory to give a prompt result to clinicians (when cultures remain in incubation), in order to speed up diagnosis for early appropriate treatment. 

The recently developed AsperGenius^®^ multiplex real-time PCR assay (PathoNostics, Maastricht, The Netherlands) allows the detection of *Aspergillus* DNA (*A. fumigatus*, *A. terreus*, and *Aspergillus* species) and mutations in the *CYP51A* gene of *A. fumigatus*. The assay consists of two PCRs: species PCR and resistance PCR. The aim of this study is to present laboratory cases of possible/proven invasive aspergillosis where this commercially available kit was applied and show that despite not being validated to tissue samples yet, this method may help in the diagnosis and decisions on therapy regimens. 

## 2. Materials and Methods

During the period 2017–2019, we applied the multiplex *Aspergillus* PCR to 12 tissue samples. In this study, we present laboratory cases where the diagnosis of aspergillosis was performed using this same assay for the detection of *Aspergillus* DNA in tissue samples, showing the usefulness of its application in these biological specimens.

Case #1 Tissue sample from the nose of a 13-year-old boy with aplastic anemia and suspicion of mucormycosis.
Case #2 Tissue sample of the bone of an immunocompetent 39-year-old man presenting extensive cutaneous ulceration of the leg with and consequent bone involvement.
Case #3 Tissue sample from the brain of a 65-year-old diabetic man who died with meningoencephalitis and suspicion of mucormycosis (under therapy with amphotericine B).
Case #4 Tissue sample (body source not referred) from a 66-year-old female from a hemato-oncology ward.

Tissue samples were collected in sterile conditions according to the protocols established by each hospital and then sent to the mycology reference laboratory within a 24 h period. Tissue specimens were sent in sterile containers with a small amount of sterile preservative-free saline solution and were divided into two pieces: one for culture and another one for PCR testing. 

Tissue fragments were sliced in smaller fragments for culture, as recommended by CLSI guidelines for the diagnosis of fungal infections [17]. A first report was given 30 days after incubation and a final report 60 days after incubation. In some cases, the sent material was small, and priority was given to what was requested by the clinician (*Aspergillus* PCR, panfungal PCR, culture); in other cases, tissue samples were sent first to other laboratories of the Portuguese NIH for bacteria or viruses PCR and the diagnosis of fungal infections was only requested further. In those cases, cultures were not performed.

Parallel to culture, DNA was extracted from fresh tissue (whenever possible, no less than 25 mg of tissue), using the High Pure PCR template preparation kit (Roche Diagnostics Corp., Indianapolis, IN, USA), according to the manufacturer’s instructions. A panfungal PCR reaction was performed in order to detect any fungal DNA present in the tissue sample. For that purpose, the universal fungal primers ITS1 (5′-TCCGTAGGTGAACCTGCGG-3′) and ITS2 (5′-GCTGCGTTCTTCATCGATGC-3′) were used to amplify DNA, as described previously [18]. Amplifications were performed in a 25 μL volume reaction of PCR beads (Illustra PuReTaq Read-to-Go; GE Healthcare, Buckinghamshire, UK), containing 15 pmol of each primer and 20–50 ng of genomic DNA.

Amplicons were purified using the ExoSAP-IT enzyme system (USB Corporation, Cleveland, Ohio), according to the manufacturer’s instructions. 

The sequencing of both strands was performed with the BigDye terminator v 1.1 cycle sequencing kit (Applied Biosystems) in the thermal cycler, using the same primers as were used in the PCR amplification. 

The resultant nucleotide sequences were edited using the program Chromas Lite v 2.01 and aligned with the program CLUSTALX v 2.1 [19]. The obtained sequences were compared with sequences deposited in the GenBank (Bethesda, MD, USA) and CBS-KNAW Fungal Biodiversity Centre (Utrecht, The Netherlands) databases in order to achieve the identification of the etiological agent. In both cases, a specific and multiplex real-time PCR directed to *Aspergillus* was performed using the AsperGenius^®^ multiplex real-time PCR assay (PathoNostics, Maastricht, The Netherlands) on the Qiagen RotorGene Q (Qiagen, Hilden, Germany) instrument, following the manufacturer’s instructions. An internal control was included to monitor inhibition or manual handling errors. The detection of mutations in the *CYP51A* gene for *A. fumigatus* conferring azole resistance was also performed using the same assay. 

## 3. Results

From the 12 tissue samples analyzed using the multiplex *Aspergillus* PCR assay, four were positive and eight negative (Table 1).

In cases #1, #2, and #4, cultures remained negative after 60 days, whereas in case #3, cultures were positive at the end of three days. The real-time PCR assay directed to *Aspergillus* gave positive signals for *Aspergillus fumigatus* (#1, #2, and #3) and *Aspergillus* sp. (#4). The results were analyzed by panfungal PCR followed by sequencing. In fact, sequencing results revealed 100% and homology with *A. fumigatus* sensu stricto (#1, #2, and #3), whereas #4 revealed 92% homology and 92% homology with *A. fumigatus* sensu stricto.

In case #1, the PCR for detection of resistance was negative with the analyzed studied locus wild type. In case #2, one mutation was detected (TR34) in the promoter of the gene *CYP51A*. This tandem repeat mutation does not confer resistance if not associated with the mutation L98H (in the gene itself), which leads to the conclusion that the *A. fumigatus* detected in the bone biopsy was susceptible to azoles. Detection of azole mutations was not performed in cases #3 and #4.

## 4. Discussion and Conclusions

PCR-based detection and identification of *Aspergillus* in tissue samples, together with the detection of possible mutations that confer azole resistance to azoles, enable the lab to give a prompt result to clinicians (while cultures remain in incubation), in order to speed up diagnosis. One problem with assessing the prevalence of resistance is noted as the recovery of *A. fumigatus* in culture is generally low and may vary considerably among different patient groups. This outcome indicates that in culture-negative patients, the presence of azole resistance will be missed [20].

An early diagnosis and prompt initiation of appropriate antifungal therapy are imperative and essential for a favorable clinical outcome. In case #1, the necrotic lesions of the nose and patient’s risk factors led to the clinical suspicion of mucormycosis. The molecular approach performed led to the rapid identification of *Aspergillus fumigatus* as the etiological agent of infection and, therefore, to a completely different antifungal therapy of the patient, with clinical improvement of the patient. In case #2, the confirmation of fungal DNA in the bone biopsy reinforced the first clinical suspicion, and antifungal therapy with azoles was maintained and the patient improved. In case #3, the clinical manifestations of meningoencephalitis with rhino-orbital involvement led to the clinical suspicion of mucormycosis; the patient was under amphotericine B therapy and died. In post-mortem analysis, an IA affecting the central nervous system was diagnosed. Amphotericine B is not the drug choice for IA, which may lead to the not-successful outcome. 

In the studied cases, there was a concordance between panfungal PCR and multiplex *Aspergillus* assay (in positive and negative cases). In case #4, the multiplex assay was not able to identify *A. fumigatus* but only *Aspergillus* sp. Panfungal PCR was positive but with low homology. The sensitivity of the primer pair for *Aspergillus* sp. is higher than the one for *A. fumigatus*, which may explain these results in a sample with low DNA yield. This *Aspergillus* multiplex assay was first developed to detect *Aspergillus* DNA from serum and BAL samples. The presented cases in our study showed the utility of its application in tissue samples. The efficacy of *Aspergillus* PCR in tissue samples was already noted by several authors [21,22]. Based on their data, PCR testing of these samples is a promising and complementary tool for identification of the underlying causative pathogen, as a positive tissue PCR result makes IA highly likely, whereas a negative result makes it rather unlikely. However, positive results do not exclude co-infection with other pathogens and the pathogens detected may not be the definite cause of disease. Therefore, a diagnosis of fungal infection should be done by the clinician, taking into account our laboratory data together with clinical data and host factors. In cases #1, #3 and #4, cultures remained negative after 60 days of incubation. Panfungal DNA followed by sequencing gave positive results and sequencing allowed the identification of *A. fumigatus* sensu strito but this is a more time-consuming methodology and does not allow the detection of mutations in the *CYP51A* gene in order to detect azole resistance. However, panfungal PCR can be very useful for diagnosing other fungi species for which there is no directed PCR. 

Molecular resistance tests have been used to assess fungal isolates as part of epidemiological surveillance studies of drug resistance and have been used to assess clinical isolates as a complement to phenotypic susceptibility testing. Real-time multiplex PCR has also been used to directly detect azole-resistant *Aspergillus* in biological samples from patients at IA risk [16]. TR34/L98H and TR46/Y121F/T289A mutations are increasingly found worldwide, and as they can be found in azole-native patients, it is important to have an assay able to detect them. Of course, its clinical utility depends on regional resistance epidemiology. In Portugal, these mechanisms of resistance were recently described [23] and it is therefore important to screen for their existence. In patients with prolonged azole therapy, a greater diversity of resistance mutations can occur and we cannot exclude other mutations. In that case, sequencing the entire *CYP51A* gene is the advisable method to detect hotspot mutations associated with azole prolonged therapy. 

Thus, the application of this assay enables the lab to give prompt results to clinicians in cases of suspicion of fungal invasive infection in order to speed up diagnosis for early appropriate treatment.

## Figures and Tables

**Table 1:** Tissue samples analyzed using the *Aspergillus* multiplex PCR assay.

Case #	Tisue Sample	*Aspergillus* Multiplex PCR	Panfungal PCR	Culture
1	Nose	*Aspergillus fumigatus*	*Aspergillus fumigatus*	Negative
2	Bone	*Aspergillus fumigatus*	*Aspergillus fumigatus*	Negative
3	Brain	*Aspergillus fumigatus*	*Aspergillus fumigatus*	*Aspergillus fumigatus*
4	Not referred	*Aspergillus* sp.	*Aspergillus fumigatus*	Negative
5	Brain	Negative	*Candida albicans*	*Candida albicans*
6	Brain	Negative	Negative	Not performed
7	Skin	Negative	Negative	*Scedosporium* sp.
8	Liver	Negative	Not performed	Not performed
9	Bone	Negative	Negative	Not performed
10	Lung	Negative	Negative	Not performed
11	Lung	Negative	Negative	Not performed
12	Sphenoid	Negative	Not performed	Not performed
